# Liver sinusoidal endothelial cell expressed vascular cell adhesion molecule 1 promotes liver fibrosis

**DOI:** 10.3389/fimmu.2022.983255

**Published:** 2022-08-25

**Authors:** Qianqian Guo, Kunimaro Furuta, Shahidul Islam, Nunzia Caporarello, Enis Kostallari, Kobe Dielis, Daniel J. Tschumperlin, Petra Hirsova, Samar H. Ibrahim

**Affiliations:** ^1^ Division of Gastroenterology and Hepatology, Mayo Clinic, Rochester, MN, United States; ^2^ Department of Gastroenterology and Hepatology, Osaka University Graduate School of Medicine, Osaka, Japan; ^3^ Department of Physiology and Biomedical Engineering, Mayo Clinic, Rochester, MN, United States; ^4^ Division of Pediatric Gastroenterology, Mayo Clinic, Rochester, MN, United States

**Keywords:** fibrosis, inflammation, liver sinusoidal endothelial cells (LSECs), hepatic stellate cells (HSCs), nonalcoholic steatohepatitis - NASH, vascular cell adhesion molecule 1 (VCAM1)

## Abstract

**Background:**

During liver injury, liver sinusoidal endothelial cells (LSECs) dysfunction and capillarization promote liver fibrosis. We have previously reported that the LSEC vascular cell adhesion molecule 1 (VCAM1) plays a key role in liver inflammation in nonalcoholic steatohepatitis (NASH) and we now aim to uncover its role in LSEC capillarization and liver fibrosis.

**Methods:**

Wild-type C57BL/6J mice were fed either chow or high fat, fructose and cholesterol diet to induce NASH and treated with either anti-VCAM1 neutralizing antibody or control isotype antibody. Inducible endothelial cell-specific Vcam1 deleted mice (*Vcam1^Δend^*) and control mice (*Vcam1^fl/fl^*) were fed choline-deficient high-fat diet (CD-HFD) to induce NASH or injected with carbon tetrachloride to induce liver fibrosis. LSECs isolated from *Vcam1^fl/fl^* or *Vcam1^Δend^* and hepatic stellate cells (HSCs) isolated from wild-type mice were cocultured in a 3-D system or a μ-Slide 2 well co-culture system.

**Results:**

Immunostaining for Lyve1 (marker of differentiated LSECs) was reduced in *Vcam1^fl/fl^* mice and restored in *Vcam1^Δend^* mice in both NASH and liver fibrosis models. Co-immunostaining showed increased α-smooth muscle actin in the livers of *Vcam1^fl/fl^* mice in areas lacking Lyve1. Furthermore, scanning electron microscopy showed reduced LSEC fenestrae in the *Vcam1^fl/fl^* mice but not *Vcam1^Δend^* mice in both injury models, suggesting that VCAM1 promotes LSEC capillarization during liver injury. HSCs profibrogenic markers were reduced when cocultured with LSECs from CD-HFD fed *Vcam1^Δend^* mice compared to *Vcam1^fl/fl^* mice. Furthermore, recombinant VCAM1 activated the Yes-associated protein 1 pathway and induced a fibrogenic phenotype in HSCs *in vitro*, supporting the profibrogenic role of LSEC VCAM1.

**Conclusion:**

VCAM1 is not just a scaffold for leukocyte adhesion during liver injury, but also a modulator of LSEC capillarization and liver fibrosis.

## Introduction

Chronic liver diseases (CLDs) of different etiologies constitute a major public health burden worldwide; there are 1.5 billion cases, accounting for approximately 1.3 million deaths each year (1, 2). Liver fibrosis is a common pathological process in CLDs that reflects advanced disease stage and represents the most important prognostic factor for morbidity and mortality (2). One of the key features of liver fibrosis is the accumulation of extracellular matrix components secreted mainly by activated hepatic stellate cells (HSCs), which are liver-specific pericytes residing in the space between the hepatocytes and the sinusoidal wall (the space of Disse). The activation of HSCs is a complex process regulated by various intercellular and paracrine signaling pathways in the liver microenvironment (3).

Liver sinusoidal endothelial cells (LSECs) are highly specialized endothelial cells lining the liver sinusoids. LSECs have a distinct morphology from vascular endothelial cells in other body organs and are characterized by the presence of pores arranged in sieve plates called fenestrae and the absence of basement membrane (4). Furthermore, growing evidence suggests that LSECs play essential roles in many pathological disorders in the liver, including metabolic dysregulation, inflammation and fibrosis (4). Given the spatial proximity between LSECs and HSCs in the liver microenvironment, LSECs under physiological conditions maintain the HSC quiescence (4). In contrast, during liver injury, LSECs lose their fenestrae and acquire a basement membrane, a phenomenon called LSEC capillarization or dedifferentiation, which is associated with HSCs activation, leading to the development of liver fibrosis (5). However, the exact molecular mediators linking LSEC capillarization to HSC activation are still unclear.

Recently, we reported that the expression of LSEC adhesion molecule vascular cell adhesion molecule 1 (VCAM1) is upregulated in murine and human NASH. We also showed that VCAM1 inhibition (both pharmacological and endothelial cell-specific genetic deletion) attenuated proinflammatory monocyte hepatic infiltration, and thereby alleviated liver fibrosis in diet-induced murine NASH models (6).

Endothelial VCAM1 binds to its cognate receptors such as integrin α9β1, α4β7 and α4β1 expressed on circulating leukocytes, leading to the firm adhesion of leukocytes to the endothelial cells, which is a critical step in the inflammatory response. Furthermore, VCAM1 functions as a signaling hub upstream of mitogen-activated protein kinase (MAPK) pathways or oxidative/nitrosative stress (7). However, it is still unclear whether VCAM1 is a hallmark of LSEC capillarization and whether VCAM1 can directly modulate HSC activation in liver fibrosis.

To answer these questions, we employed two mouse models known to induce significant liver fibrosis together with pharmacological blockade, or endothelial cell-specific knockout of VCAM1. We also treated human HSCs with recombinant VCAM1; in addition, we employed 2-D and 3-D co-culture systems of LSECs and HSCs to mimic the liver microenvironment and show that endothelial VCAM1 promotes LSEC capillarization and liver fibrosis during liver injury.

## Methods

### Materials

Palmitate (PA) (P0500) was obtained from Sigma-Aldrich (St. Louis, MO). LPC (Sigma, St. Louis, MO) was dissolved as previously described (8). Primary antisera employed for the studies include anti-alpha smooth muscle actin (αSMA) (ab124964) and anti-fibronectin (ab2413) antibodies from Abcam (Cambridge, MA), anti-GAPDH (MAB374) from Millipore Sigma, anti-β-actin (sc-47778) from Santa Cruz Biotechnologies (Santa Cruz, CA) and anti-Lyve1 (AF2125) from R&D systems (Minneapolis, MN), anti-F4/80 (70076) and anti-phospho Yap (Ser127) (4911) from Cell Signaling Technology (Danvers, MA), anti-Yap1 (sc-101199) from Santa Cruz Biotechnologies (Santa Cruz, CA), anti-type I collagen antibody (1310-01) from Southern Biotech. Recombinant human VCAM1 (rhVCAM1) (862-VC) was obtained from R&D systems.

### Cells

Primary human liver sinusoidal endothelial cells (hLSECs) were purchased from ScienCell Research Laboratories (#5000, San Diego, CA). Primary mouse LSECs were isolated using a method based on liver collagenase perfusion and immunomagnetic selection as previously described (6, 9). Briefly, liver was digested with collagenase infused *via* portal vein and obtained cell suspensions were centrifuged at 50 g for 2 minutes to remove hepatocytes. The supernatant, which includes non-parenchymal cells was subjected to LSEC isolation using CD146 MicroBeads (Miltenyi Biotec, Bergisch Gladbach, Germany) following the manufacture’s instruction. hLSECs and primary mouse LSECs were cultured in Endothelial Cell Growth Medium (#211-500, Sigma) consisting of 5% fetal bovine serum (FBS), 1% endothelial cells growth supplement, and 1% primocin (InVivoGen, San Diego, CA) solution. Mouse primary hepatic stellate cells (mHSCs) were isolated as described previously (10). Briefly, the isolation of HSCs from mouse liver is composed of three subsequent steps: (a) *In situ* pronase/collagenase perfusion of mouse liver; (b) subsequent *in vitro* digestion; and (c) density gradient-based separation of HSCs from other hepatic cell populations. mHSCs were cultured in complete media DMEM (Life Technologies #11965092), 10% FBS and 1% primocin (InVivoGen, San Diego, CA) solution. Primary human hepatic stellate cells (hHSCs, ScienCell Research Laboratories #5300) were cultured in complete stellate growth medium (ScienCell Research Laboratories #5301) containing 1% primocin. Both hLSECs and hHSCs were maintained according to the manufacturer’s instructions and only the cells with a passage number of 3 or 4 were used for the experiments. All the cell cultures were maintained at 37°C in a humidified atmosphere of 5% CO2.

### 3-D co-culture of primary LSECs and HSCs

Endothelial cell growth basal medium was combined with Matrigel Matrix (Corning, NY, USA, Cat# 356231) in a 3:2 ratio to produce a 40% Matrigel solution. Each well of a 96-well culture plate was coated with 80 µl of the 40% Matrigel solution. The Matrigel layers were then incubated at 37°C for 45 minutes to enhance polymerization. 1x10^5^ mouse LSECs isolated from CD-HFD-fed *Vcam1^fl/fl^* or *Vcam1^Δend^* mice and 1x10^5^ mouse HSCs were suspended in endothelial cell growth medium and seeded on the freshly solidified Matrigel layer. Co-culture of hLSECs and hHSCs was performed in the same setting after the pretreatment of hLSECs with or without PA for 16 hours. After 3 days, cells were recovered from Matrigel using Corning Cell Recovery Solution (Corning, Cat#35425), and total RNA extraction, cDNA synthesis, and qPCR analysis were performed.

### 2-D co-culture of primary hLSECs and hHSCs

hLSECs and hHSCs were co-cultured using μ-Slide 2 well Co-culture (ibidi, Lochhamer, Germany). Briefly, 1x10^4^ hLSECs were seeded in the peripheral wells and 1x10^4^ hHSCs were seeded in the central well, then the cells were cultured overnight. hLSECs were treated with LPC 20 μM, after 4 hours LPC containing medium was removed and replaced with LSEC complete medium to fill up the central and the peripheral wells and the inter-cellular communication between hLSECS and hHSCs *via* soluble factors was examined. After 6 hours of co-culture, hHSCs were processed for αSMA immuno-staining following the protocols described in the immunocytochemistry section.

### Immunocytochemistry and confocal microscopy

hHSCs were treated or co-cultured with hLSECs as desired and fixed with 4% PFA for 20 min at room temperature, and permeabilized using 0.01% TritonX-100 for 5 min, then blocked with 5% BSA for one hour. Cells were incubated with primary antibodies, anti-αSMA (1:250), or anti-Yap1 (1:500) overnight at 4°C. Cells were labeled using Alexa Fluor 596-conjugated donkey anti-rabbit IgG (1:2,000), Alexa Fluor 488-conjugated donkey anti-rabbit IgG (1:2,000), or Alexa Fluor 596-conjugated chicken anti-mouse IgG (1:2,000), and observed under confocal microscopy (LSM 980, Zeiss, Jena, Germany). 4’, 6-diamidino-2-phenylindole (DAPI) was used for the nuclear counterstain. ZEN 2.3 lite software (ZEISS) was used for acquiring images.

### Primary human hepatic stellate cell activation assay

To culture primary hHSCs on a matrix that simulates the liver stiffness *in vivo*, CytoSoft 6-well plates with a rigidity of 0.2 kPa were obtained from Advanced Biomatrix (Cat# 5165, Carlsbad, CA), these plates have a 0.5 mm thick silicone gel in each well. Before seeding the cells, the wells were coated with 0.1 mg/ml PureCol Type I collagen solution (Cat# 5005, Advanced Biomatrix INC, Carlsbad, CA) to allow cell attachment. Primary HSCs were plated in a CytoSoft 6-well plate at a concentration of 3 x 10^5^ cells/well. After serum starvation overnight, the HSCs were treated with 0.5 μM of rhVCAM1 for 48 hours. Cells were then harvested for RT-PCR analysis.

### Immunoblot analysis

Cells were lysed using RIPA buffer (50 mM Tris-HCl, pH 7.4; 1% Nonidet P-40; 0.25% sodium deoxycholate; 150 mM NaCl; 1 mM EDTA with protease inhibitors) followed by centrifugation at 15,000g for 15 min at 4°C. Protein concentrations of the lysates were measured by the Bradford assay method (Sigma-Aldrich). Equal amount of protein was loaded onto Sodium dodecyl sulfate (SDS)-Polyacrylamide gel electrophoresis (PAGE) gels, transferred to nitrocellulose membrane (Bio-Rad, Hercules, CA) and incubated overnight with the primary antibody of interest. All primary antibodies were used at a dilution of 1:1,000 unless otherwise recommended by the manufacturer. Horseradish peroxidase-conjugated secondary antibodies against rabbit (Alpha Diagnostic International, San Antonio, TX) or mouse (Southern Biotech, Birmingham, AL) were used at a dilution of 1:5,000 and incubated for 1 hour at room temperature. Proteins were detected using enhanced chemiluminescence reagents (GE Healthcare, Chicago, IL). GAPDH protein levels were used as loading controls.

### Animals

Study protocols were conducted as approved by the Institutional Animal Care and Use Committee (IACUC) of Mayo Clinic. The methods employed in the current study were conducted in accordance with IACUC guidelines for the use of anesthetics in experimental mice. Mice were housed and bred in a temperature-controlled 12:12-hour light-dark cycle facility with free access to diet. All interventions occurred during the light cycle. C57BL/6J mice were purchased from Jackson Laboratory (Bar Harbor, ME).

### Generation of endothelial cell specific Vcam1 knockout mice

*Vcam1^fl/fl^* mice on the C57BL/6J background (Jackson Laboratory, Cat. 007665) were crossed with a line expressing tamoxifen-inducible Cre recombinase (CreERT2) under the regulation of the vascular endothelial cadherin (VE-Cadherin) promoter (Cdh5(PAC)-CreERT2) (11, 12), and the offspring *Vcam1^fl/fl^ Cdh5(PAC)-CreERT2* mice were obtained. At 6 weeks of age, *Vcam1^fl/fl^ Cdh5(PAC)-CreERT2* mice were injected intraperitoneally with 4 mg of tamoxifen for 5 consecutive days and used as endothelial cell-specific Vcam1 knockout mice (referred to as *Vcam1^Δend^*). Littermates that do not have the *Cdh5(PAC)-CreERT2* transgene (referred to as *Vcam1^fl/fl^*) received the same tamoxifen dose and served as control mice.

### Diet-induced murine NASH models

C57BL/6J wild-type (WT) mice were fed either a chow diet (5053 PicoLab Rodent Diet 20, LabDiet, St Louis, MO) or a diet rich in fat, fructose, and cholesterol (FFC) starting at the age of 8-weeks for 24 weeks. FFC diet consists of 40% energy as fat (12% saturated fatty acid, 0.2% cholesterol) (AIN-76A Western Diet, TestDiet, St Louis, MO), with fructose (23.1 g/L) and glucose (18.9 g/L) in the drinking water. The FFC diet phenocopies the metabolic and histological features of the human NASH (13), and has been extensively validated (14, 15). At 20 weeks on the diet, the mice were randomized to receive either anti-VCAM1 neutralizing antibody (M/K-2.7), (Genetex, GTX14360) or IgG isotype antibody (BE0088, InVivoMAb). Mice were injected with 10 mg/kg body weight of either the antibodies or IgG isotype intraperitoneally, twice per week for the last 4 weeks of the feeding studies. In an independent study, *Vcam1^fl/fl^* and *Vcam1^Δend^* mice were fed the choline-deficient high-fat diet (CD-HFD) (Research Diet, Cat. A06071302), which consists of 60% fat, 0.1% methionine, and no added choline, starting at 8 weeks of age for 6 weeks. Mice fed the CD-HFD experienced minimal body weight loss compared to the traditional choline-deficient diet and had hepatic steatosis, ALT elevation, hepatocytes ballooning, hepatic inflammation, and fibrosis, recapitulating the histological features of human NASH as we and others have shown in previous studies (6, 16).

### Liver fibrosis model

Liver fibrosis was induced by intraperitoneal injection of carbon tetrachloride (CCl4), 1 µL/g of body weight, (Sigma-Aldrich #319961) into *Vcam1^Δend^* and *Vcam1^fl/fl^* mice, twice a week for 4 weeks. Mice were sacrificed 48 hours after the last injection.

### Histology, immunohistochemistry, and digital image analysis

Formalin-fixed paraffin-embedded mouse liver tissue sections were deparaffinized, hydrated, and stained with antibody against Lyve-1 (1:100), F4/80 (1:500), or αSMA (1:1,000). The bound antibody was detected using a Vectastain ABC kit for goat (PK-6105, Vector Laboratories, Burlingame, CA) or DakoEnVision+Dual Link System-HRP kit (#K4063) and DAB substrate (Vector Laboratories) according to the manufacturer’s instructions; the tissue sections were counterstained with hematoxylin. Lyve-1/F4/80/αSMA positive areas were quantified by digital image analysis of 10 random fields per slide per animal using ImageJ software. For the co-staining study, primary antibodies against Lyve-1 (1:250) and αSMA (1:500) were detected using Alexa Fluor 488-conjugated chicken anti-goat IgG (A21467) and Alexa Fluor 596-conjugated donkey anti-rabbit IgG (Thermo Fisher Scientific), respectively, and then the co-stained liver tissues were examined by confocal microscopy (LSM 980, Zeiss, Jena, Germany). 4’, 6-diamidino-2-phenylindole (DAPI) was used for the nuclear counterstain. ZEN 2.3 lite software (ZEISS) was used for acquiring images.

### SEM study and analysis of LSEC fenestration

Mice were injected *via* the portal vein with saline and then Trump’s fixative consisting of 4% formaldehyde and 1% glutaraldehyde in phosphate buffered saline PBS (pH 7.2) to fix the liver in situ. Fixed livers were removed and cut into 2 mm^2^ size and immersed in Trump’s fixative at 4°C overnight. The specimens were dehydrated in a graded series of ethanol, and dried. Subsequently, the sections were coated with a thin layer of platinum/palladium and visualized under an S-4700 electron microscope (Hitachi Inc, Pleasanton, USA). The numbers of fenestrae in LSECs in randomly selected 5 fields per mouse were quantified using imageJ software as previously described (17).

### Quantitative real-time PCR

Total RNA was isolated with the RNeasy Mini Kit (Qiagen, Valencia, CA) and was reverse transcribed with moloney murine leukemia virus reverse transcriptase and oligo-dT random primers (both from Invitrogen, CA, USA). Quantification of gene expression was performed by real-time PCR using SYBR green fluorescence on a QuanStudio 6 Flex (Applied biosystems, Thermo Fisher Scientific, USA). Target gene expression was calculated using the ΔΔCt method and was normalized to *18S* or *Gapdh* mRNA expression levels, which were stable across experimental groups. Target genes primers sequences used in this study were shown in **Table 1 in Supplementary Materials**.

**Figure 1:**
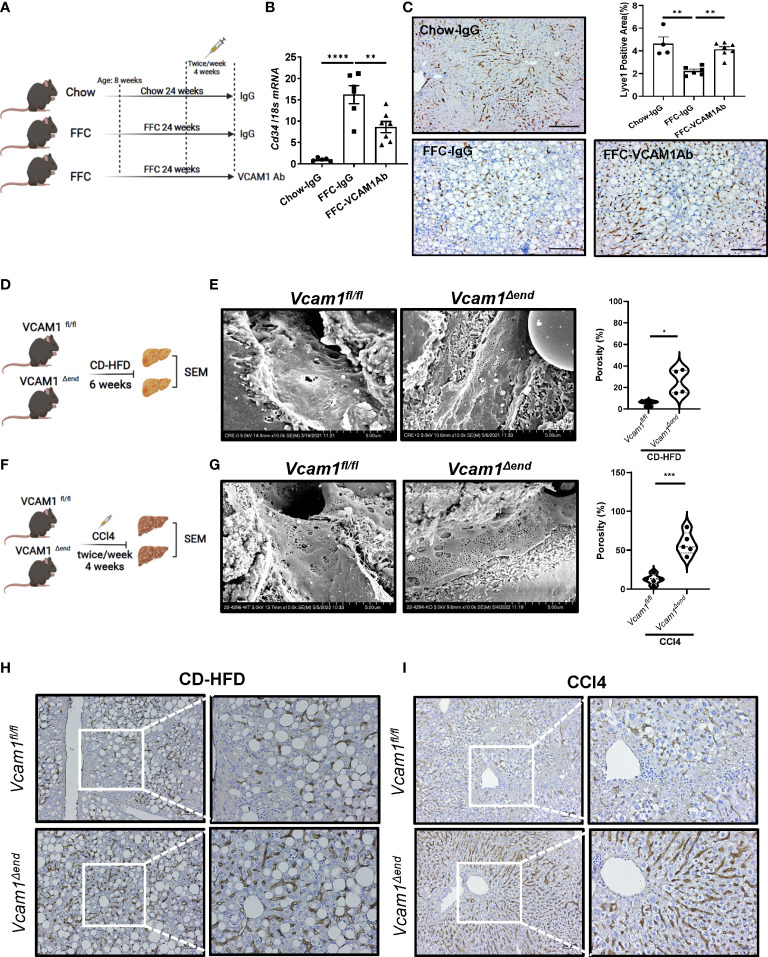
Endothelial VCAM1 promotes LSEC capillarization during liver injury. Eight-week-old WT C57BL/6J mice were fed either chow or FFC diet for 24 weeks to induce NASH and treated with either anti-VCAM1Ab or control IgG isotype Ab (IgG) twice a week for the last 4 weeks. **(A)** Schematic representation of the experimental mouse study. **(B)** Hepatic mRNA expression of Cd34 was assessed by real-time PCR. Fold change was determined after normalization to 18s rRNA and expressed relative to chow-IgG mice. n=5-7. **(C)** Representative images of Lyve1 immunostaining of liver sections (left). Scale bar: 100 μm. Lyve1 positive areas were quantified in 10 random 10x microscopic fields and averaged for each animal (right). n=4-7. Vcam1^fl/fl^ and Vcam1^Δend^ mice were fed the CD-HFD diet starting at the age of 8 weeks for 6 weeks to induce NASH. **(D)** Schematic representation of the experimental mouse study. **(E)** Representative SEM images of the mouse livers. Scale bar: 5 μm (left) as shown on the bottom of the picture. The frequency of fenestrae was presented as porosity and quantified using image J (right). Eight-week-old Vcam1^Δend^ mice and Vcam1^fl/fl^ mice were treated with CCl4 intraperitoneally (1μL/g body weight), two time a week for 4 weeks to induce liver fibrosis. **(F)** Schematic representation of the experimental mouse study. Vcam1^fl/fl^ and Vcam1^Δend^ mice were injected intraperitoneally with CCl4 twice a week for 4 weeks to induce liver fibrosis. **(G)** Representative SEM images of the mouse livers. Scale bar: 5 μm (left) as shown on the bottom of the picture. The frequency of fenestrae was presented as porosity and quantified using image J (right). Representative images of Lyve1 immunostaining of liver sections from CD-HFD induced NASH mice **(H)** or CCl4 induced liver fibrosis mice **(I)**. Scale bar: 50 μm. *, **, ***, **** indicate statistical significance with p < 0.05, p < 0.01, p < 0.001 and p < 0.0001, respectively.

To determine the relevance of VCAM1 in the deleterious LSEC phenotype caused by chronic liver injury *in vivo*, we first examined whether pharmacological blockade of VCAM1 could prevent sinusoidal capillarization, a hallmark of injurious and pro-fibrogenic phenotype observed in LSECs. To this end, we employed a mouse model of NASH (**Figure 1A**) induced by a high fat, fructose, and cholesterol (FFC) diet that recapitulates the clinical and histological features of the human disease (1, 2). In this model, the mRNA level of the capillarized endothelial cell marker *Cd34* was increased in the FFC-fed control antibody (IgG)-treated mice and reduced with VCAM1 neutralizing antibody (VCAM1Ab) treatment (**Figure 1B**). In line with this data, immunostaining showed that the differentiated LSEC marker Lyve1 was reduced by FFC feeding and restored with VCAM1Ab treatment (**Figure 1C**). VCAM1 is expressed by immune cells and cholangiocytes in addition to LSEC (3, 4). Hence, we next employed our inducible Cre-mediated endothelial cell-specific *Vcam1* knockout mice to investigate the role of VCAM1 expressed on LSECs in sinusoidal capillarization during NASH. Control mice (*Vcam1^fl/fl^*) and knockout mice (*Vcam1^Δend^*) were fed choline-deficient high-fat diet (CD-HFD) to induce NASH (**Figure 1D**) (5, 6). As shown in **Figure 1E**, scanning electron microscopy (SEM) of liver sections demonstrated the LSEC fenestrae from both groups of mice and quantified by porosity showing restoration of fenestrae in CD-HFD-fed *Vcam1^Δend^* mice compared to *Vcam1^fl/fl^* mice.

We next examined whether deletion of LSEC *Vcam1* also attenuates LSEC capillarization in the CCl4-induced chronic liver injury model (**Figure 1F**). Interestingly, SEM of liver sections from *Vcam1^Δend^* mice showed restoration of the LSEC fenestrae (**Figure 1G**). Capillarized LSECs are often identified in chronic liver disease and advanced liver fibrosis (7). Indeed, immunostaining of Lyve1 was reduced significantly in *Vcam1^fl/fl^* mice from both CD-HFD induced NASH model and CCl4-induced liver fibrosis model (**Figure 1H, I** and **Supplementary Figure 1A, B**) and restored in *Vcam1^Δend^* mice. Interestingly, double immunofluorescent staining showed significant reduction of Lyve1 in CD-HFD fed *Vcam1^fl/fl^* mice when compared to chow-fed *Vcam1^fl/fl^* mice, especially in areas with high αSMA expression. Likewise, restoration of Lyve1 and reduction of αSMA expression and liver fibrosis was observed in CD-HFD fed *Vcam1^Δend^* (**Supplementary Figure 1C**). These data suggest the involvement of LSEC capillarization in HSC activation. Taken together, these findings imply that during liver injury, endothelial VCAM1 promotes hepatic sinusoidal endothelial cell capillarization and may contribute to the loss of HSC quiescence.

### LSECs under toxic lipid treatment promote HSCs activation *via* a VCAM1 dependent mechanism

**Figure 2:**
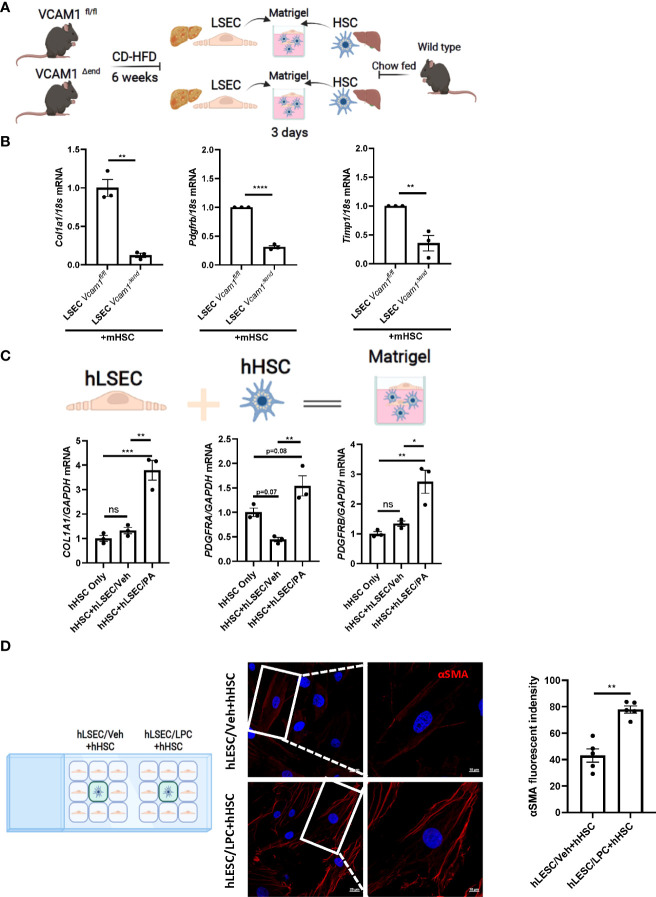
Lipotoxic LSECs activate HSCs during NASH in a VCAM1 dependent manner. Primary LSECs were isolated from Vcam1^Δend^ or Vcam1^fl/fl^ mice with NASH and primary HSCs were isolated from healthy wild type mice and co-culured using 3-D co-culture sytem. **(A)** Schema of the 3-D coculture system used for mouse primary cells co-culture. **(B)** mRNA levels of HSCs activation markers, Col1a1, Pdgfrb and Timp1 were assessed by real-time PCR. Fold change was determined after normalization to 18s rRNA. n=3. **(C)** Human primary LSECs and HSCs were co-cultured using the same 3-D co-culture system. hLSECs treated with vehicle or palmitate (PA) 500 μM overnight then co-cultutred with hHSCs for 3 days in LSEC growth medium without PA treatment, HSCs activation was examined by mRNA expression of COL1a1, PDGFRA and PDGFRB. n=3. **(D)** hLSECs and hHSCs were co-cultured using a 2-D co-culture μ-slide to examine the effect of hLSECs-derived soluble factors on hHSCs activation. hLSECs in the peripheral wells were pre-treated with LPC 20μM for 4 hours, then LPC containing media was replaced and filled up to allow for intercellular communication via soluble factors. After 6-hours of co-culture, hHSCs activation was examined by αSMA staining. Scale bar: 20μm left pannel, 10μm right pannel. αSMA fluorescent density from 5 random fields was quantified using ImageJ software. *, **, ***, **** indicate statistical significance with p < 0.05, p < 0.01, p < 0.001 and p < 0.0001, respectively. Statistically non-significant results were labeled as ns.

Lipotoxicity secondary to excess circulating saturated free fatty acids (SFA)-induced cellular stress is a major driver of NASH pathogenesis. LSEC VCAM1 expression is enhanced in mice and human with NASH and upregulated upon treatment with SFA palmitate *in vitro* (5, 8). To investigate whether LSECs VCAM1 can directly activate HSCs and mimic the spatial proximity of LSECs and HSCs in the liver microenvironment, we employed a matrigel-based 3-D co-culture system consisting of these two cell types (**Figure 2A**). We co-cultured primary LSECs isolated from CD-HFD-fed *Vcam1^fl/fl^* and *Vcam1^Δend^* mice with primary HSCs isolated from chow-fed wild-type mice. mRNA expression of the pro-fibrogenic markers *Col1a1, Pdgfrb and Timp1* in HSCs co-cultured with NASH liver-derived *Vcam1^Δend^* LSECs was reduced when compared to HSCs co-cultured with NASH liver-derived *Vcam1^fl/fl^* LSECs (**Figure 2B**). We next employed primary human cells and examined whether PA-primed LSECs promote the induction of stellate cell activation using the same 3-D co-culture system. We have previously demonstrated that the primary human LSECs used for these experiments had well preserved LSEC-specific features such as higher expression of Lyve-1 and Stabilin-2 compared to human umbilical vein endothelial cells (HUVECs) (9). We also confirmed that the primary human HSCs showed increased protein expressions of the extracellular matrix component fibronectin and the HSC activation marker α-SMA, suggesting that these cells are genuine quiescent HSCs rather than activated fibroblasts (**Supplementary Figure 2A**) as previously described by us in details (10, 11). When co-cultured with PA-primed hLSECs, hHSCs showed significant increase in *COL1A1*, *PDGFRA* and *PDGFRB* gene expressions compared to those co-cultured with the control non-treated hLSECs (**Figure 2C**).

To investigate whether cell contact is required for the LSEC-induced HSC activation, we employed a 2-D co-culture of hLSECs and hHSCs using μ-slide system to examine the potential role of soluble VCAM1 released from LSECs under toxic lipid treatment in HSC activations. VCAM1 is known to be cleaved by a disintegrin and metalloproteinase 17 (ADAM17) at the extracellular site proximal to the cell membrane and the released free form is biologically active (18). In this co-culture system, both cell types were seeded in separate minor wells, but can only communicate by medium. We employed a well validated lipotoxic agent lysophosphatidylcholine (LPC) (12, 13) When co-cultured with LPC-treated hLSECs, hHSCs displayed enhanced activation as shown by enhanced αSMA staining (**Figure 2D**) compared to hHSCs co-cultured with non-treated hLSECs. Taken together these data suggest that during lipotoxicity, LSEC-derived VCAM1 in a free form enhances hepatic stellate cell activation thereby potentiates the development of liver fibrosis.

### Hippo pathway effector Yap1 is involved in VCAM1 mediated HSCs activation

**Figure 3:**
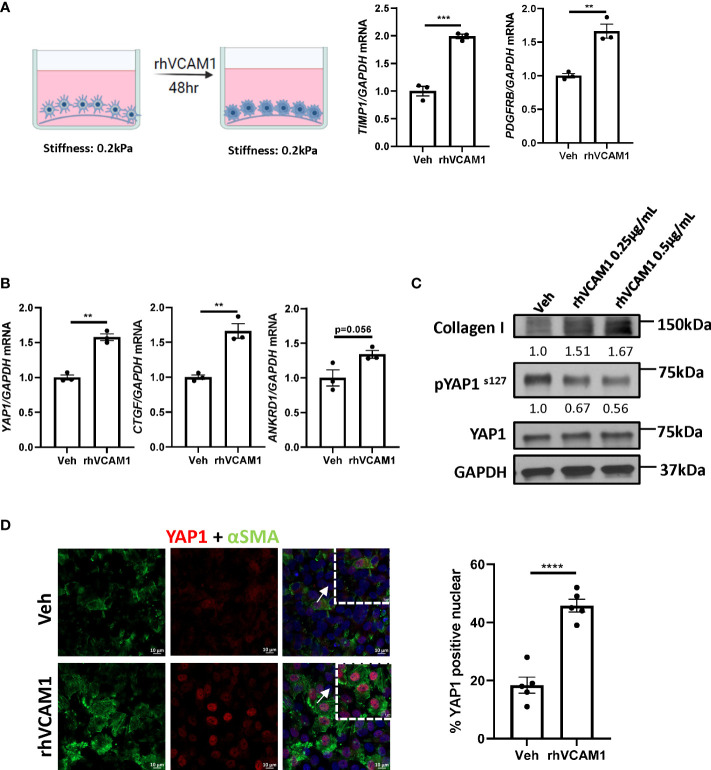
VCAM1-induced HSC activation is YAP1-dependent. Primary human HSCs were cultured using a cyto-soft plate with 0.2 kPa stiffness. hHSCs were treated with vechicle, 0.25 μM or 0.5 μM rhVCAM1 for 48 hours. **(A)** hHSCs activation was determined by mRNA levels of TIMP1 and PDGFRB. n=3. **(B)** Activation of hippo pathway protein YAP1 as well as its targets CTGF and ANKRD1 were examined by mRNA expression. n=3. **(C)** hHSCs were treated with rhVCAM1 for 48 hours. COL1A1 and phosphorylated and total YAP1 protein expressions were determined by western blotting. GAPDH was used as a loading control. The optical density of the bands (normalized to GAPDH for collagen 1 and to total YAP1 for phosphorylated YAP1) were quantified using ImageJ software and indicated below each band. **(D)** hHSCs were treated with vechicle or rhVCAM1 0.5 μM for 5 days, YAP1 subcellular locolization was examined by confocal microscopy and immunofluorescence using an anti-YAP1 antibody (red), HSCs activation was accessed by aSMA immunofluorescence (green). YAP1 positive nuclei were quantified from 5 random fields using imageJ software. Scale bar: 10μm. **, ***, **** indicate statistical significance with p < 0.01, p < 0.001 and p < 0.0001, respectively.

To avoid the spontaneous activation of HSCs by culturing on the plastic plate surface, we utilized the soft silicon culture surface with rigidity of 0.2 kPa, which simulates a healthy liver stiffness. To demonstrate that lipotoxic LSECs can promote HSC activation in a VCAM1-dependent manner, we treated hHSCs with recombinant human VCAM1 (rhVCAM1) and identified significant upregulation of the mRNA expression of HSC activation markers *TIMP1* and *PDGFRB* when compared to control cells (**Figure 3A**). These data indicate that VCAM1 has the potential to activate quiescent HSCs. Next, we aimed to identify the potential regulatory mechanism underlying LSEC VCAM1-induced HSC activation. Hippo pathway and its downstream effector Yes-associated protein 1 (YAP1) is a known regulator of HSC activation, especially during the early stage of liver injury (14). Interestingly, recombinant human VCAM1-treated hHSCs showed significant induction of *YAP1* as well as its target genes including connective tissue growth factor (*CTGF*) and ankyrin repeat domain protein 1 (*ANKRD1*) (**Figure 3B**). Furthermore, when treated with rhVCAM1 protein for 48 hours, hHSCs showed decreased phosphorylation of Yap1 as well as slight increase of total Yap1 (**Figure 3C**). Likewise, rhVCAM1-treated hHSCs displayed increased nuclear YAP1 when compared with vehicle-treated cells (**Figure 3D**). We next examined the correlation of the activation of YAP1 protein and αSMA expression in HSCs and showed that rhVCAM1 treated HSCs also displayed enhanced activation as shown with αSMA staining (**Figure 3D**) compared to vehicle-treated cells. Collectively, these data suggest that VCAM1 induced HSC activation is likely mediated by a YAP1 dependent mechanism.

### Endothelial cell-specific loss of VCAM1 ameliorates liver fibrosis in mice with CCl4-induced liver injury

**Figure 4:**
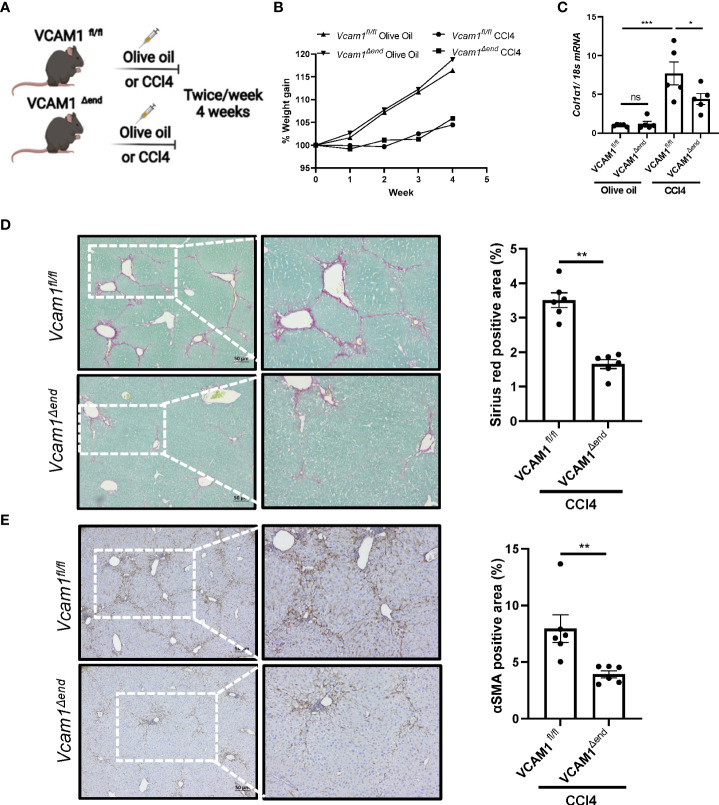
Endothelial cell-specific loss of VCAM1 is protective during liver fibrosis. Eight-week-old Vcam1^Δend^ mice and Vcam1^fl/fl^ mice were treated with either olive oil or CCl4 intraperitoneally (1μL/g body weight), twice a week for 4 weeks to induce liver fibrosis. **(A)** Schematic representation of the experimental mouse study. **(B)** Animal growth curve presented by weight gain during olive oil/CCl4 induction. Liver fibrosis was assessed by **(C)** mRNA expression of Collagen1a1, **(D)** Sirius red staining and **(E)** immunostaining of αSMA. Scale bar: 50μm, n=3-6. *, **, ***, indicate statistical significance with p < 0.05, p < 0.01 and p < 0.001, respectively. Statistically non-significant results were labeled as ns.

To examine the profibrogenic role of LSEC VCAM1 *in vivo*, we employed the CCl4 experimental liver fibrosis mouse model (**Figure 4A**). CCl4 treatment was well tolerated in mice from the different experimental groups and did not affect weight gain (**Figure 4B**). CCl4-treated *Vcam1^fl/fl^* mice showed increased *Col1a1* mRNA expression in liver when compared to olive oil-treated group mice. In contrast, CCl4-treated *Vcam1^Δend^* mice had a significant reduction in *Col1a1* mRNA levels (**Figure 4C**). Likewise, when compared to CCl4-treated *Vcam1^fl/fl^* mice, *Vcam1^Δend^* had reduced liver fibrosis when assessed by Sirius red staining, and αSMA immunostaining (**Figure 4D, E**). Collectively, these data support the profibrogenic role of LSEC VCAM1 in liver injury.

### Endothelial cell-specific loss of VCAM1 ameliorates liver inflammation in mice with CCl4-induced liver injury

**Figure 5:**
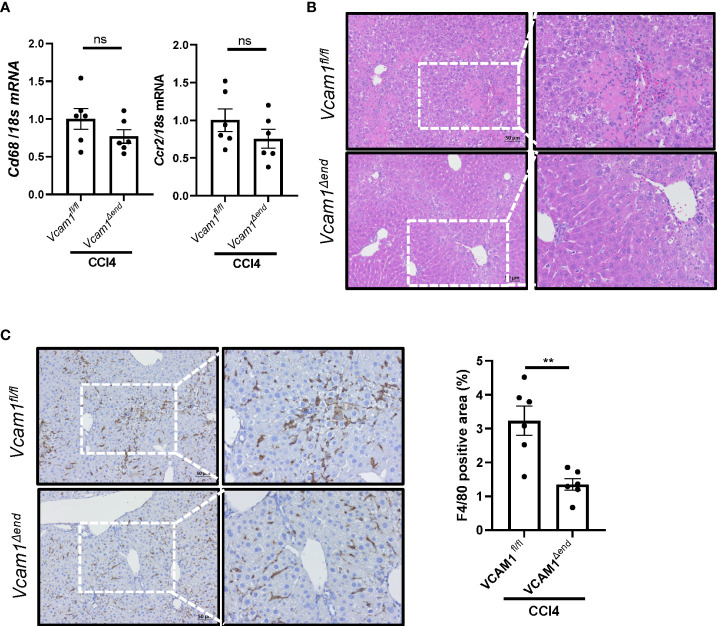
Endothelial cell-specific loss of VCAM1 is protective against inflammation during liver fibrosis. Eight-week-old Vcam1^Δend^ mice and Vcam1^fl/fl^ mice were treated with either olive oil or CCl4 intraperitoneally (1μL/g body weight), twice a week for 4 weeks. Liver inflammation was assessed by **(A)** Cd68 and Ccr2 mRNA expression, **(B)** H&E staining and **(C)** F4/80 immunostaining. Scale bar: 50μm, n=3-6. ** indicate statistical significance with p < 0.01. Statistically non-significant results were labeled as ns.

Given the known role of VCAM1 in immune cell adhesion and liver inflammation in NASH (5), we sought to assess liver inflammation in CCl4-treated *Vcam1^Δend^* mice, and showed reduced inflammatory infiltrate as assessed by H&E staining (**Figure 5B**) in these mice as compared to the *Vcam1^fl/fl^* mice. Furthermore, IHC staining for the macrophage specific marker F4/80, showed reduced immunostaining in CCl4-treated *Vcam1^Δend^* mice compared to CCl4-treated *Vcam1^fl/fl^* mice (**Figure 5C**), although the changes of *Cd68* or *Ccr2* mRNA expressions were not statistically significant between the groups (**Figure 5A**). These findings suggest that CCl4-treated *Vcam1^Δend^* mice showed attenuated liver inflammation compared to CCl4-treated *Vcam1^fl/fl^* mice, which is consistent with its known role in immune cell adhesion and hepatic infiltration during liver injury. However, the difference in CCl4-induced liver inflammation between *Vcam1^fl/fl^* and *Vcam1^Δend^* mice was relatively modest, when compared with the striking reduction in liver fibrosis in the CCl4-treated *Vcam1^Δend^* mice, suggesting that LSEC-expressed VCAM1 promotes liver fibrosis also through direct interaction with hepatic stellate cells..

## Discussion

**Figure 6:**
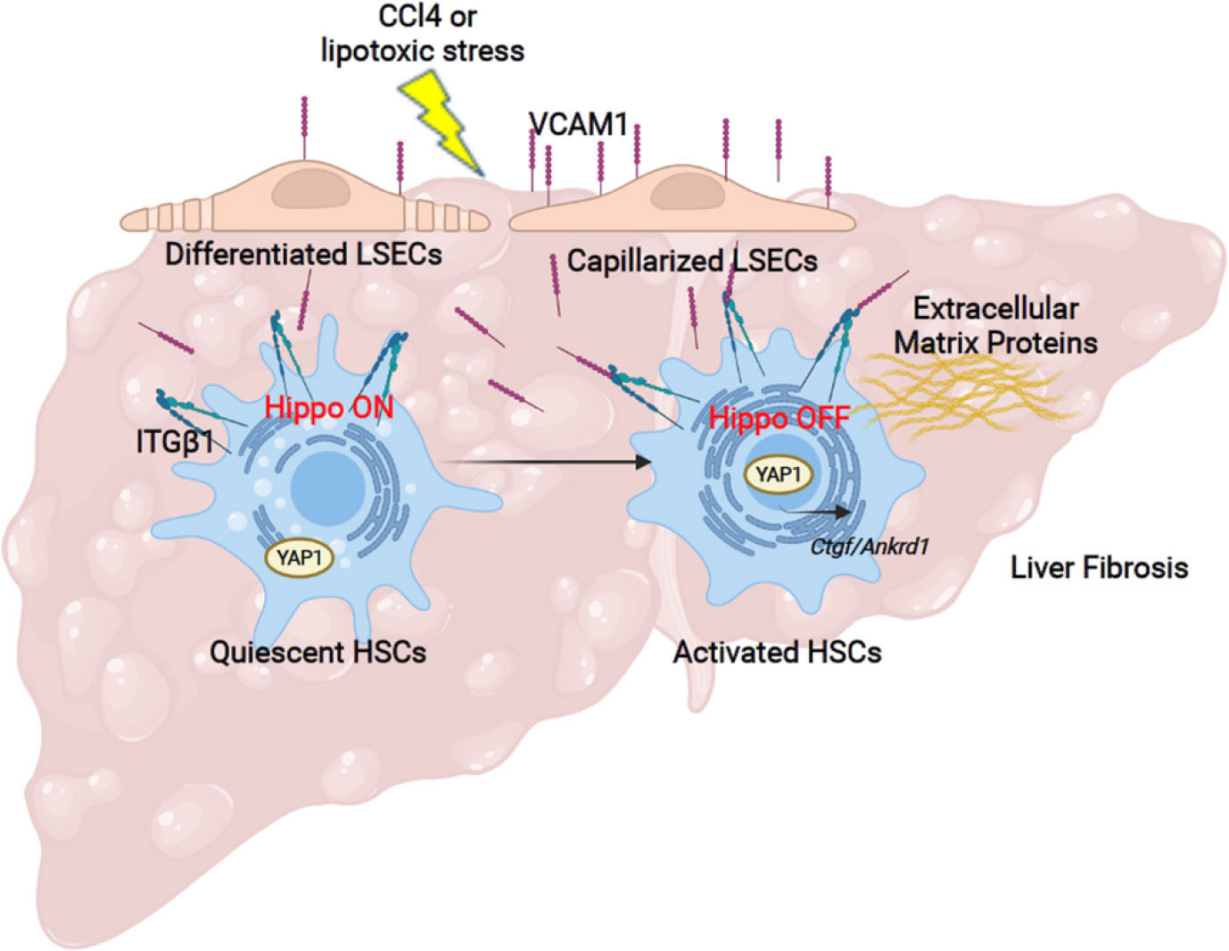
Liver sinusoidal endothelial cell expressed VCAM1 promotes hepatic stellate cells activation via Hippo pathway target protein Yap1 activation. Schematic representation of the major findings of the study showing a direct interaction between LSEC VCAM1 and hepatic stellate cells, resulting in hepatic stellate cell activation likely by a YAP1 dependent pathway.

The principal findings of the present study provide mechanistic insights regarding the role of the adhesion molecule VCAM1 expressed on LSECs in the development of liver fibrosis (**Figure 6**). Our results indicate that i) LSEC VCAM1 promotes endothelial capillarization in two murine models of chronic liver injury, ii) toxic lipid-induced exuberant expression of VCAM1 promotes HSC activation *via* YAP1 signaling pathway, iii) endothelial cell-specific deletion of VCAM1 ameliorates CCl4-induced mouse liver fibrosis. Our findings are discussed in greater details below.

Since its discovery as an endothelial cell surface glycoprotein, VCAM1 has been recognized for its essential roles in leukocyte adhesion *via* its cognate binding with counterpart adhesion molecules on the leukocyte surface such as integrin α_4_β_1_ and α_4_β_7_ (15, 16). Given its role as a foothold for leukocyte recruitment, VCAM1 has been implicated in the pathogenesis of numerous inflammatory diseases including rheumatoid arthritis, asthma and atherosclerosis (17, 19, 20). Likewise, we recently reported that in NASH pathogenesis, toxic lipid-induced aberrant expression of LSEC VCAM1 mediates hepatic recruitment of pro-inflammatory monocytes, thereby promoting inflammation and fibrosis in the liver (5). On the other hand, HSCs are known to be activated by various stimuli including direct interaction or paracrine signaling from LSECs (21, 22). Moreover, recent single cell studies delineated inferred ligand-receptor interactions between LSECs and HSCs during liver fibrosis both in mouse and humans (18, 23). However, the role of LSEC derived factors in the evolution of liver fibrosis during liver injury is an area ripe for further investigation. Hence, the present study shows a critical role of LSEC VCAM1 in HSC activation and is consistent with previous human studies that indicate that serum levels of soluble VCAM1 can predict liver fibrosis severity in NAFLD patients (24).

LSECs are distinguished from other endothelial cells in the body by the lack of basement membrane and the presence of fenestrae regularly arranged in so-called sieve plates. Differentiated LSECs (LSECs under physiological conditions) can maintain HSC quiescence in a paracrine manner (7, 25). During chronic liver injury of various etiologies, LSECs lose their quiescent phenotype (fenestrae), a phenomenon called ‘capillarization’, which ensues prior to liver fibrosis (26). Indeed, capillarized or dedifferentiated LSECs can promote HSC activation and liver fibrosis (25). However, the exact mechanism of LSEC capillarization in chronic liver disease is largely obscure. Several mechanisms have been implicated in LSEC capillarization thus far, such as vascular endothelial growth factor (VEGF) pathway, Notch signaling pathway and hedgehog signaling pathway (25, 27, 28). Other studies attributed the development and loss of fenestrae in LSECs to actin-mediated cytoskeletal reorganization (29, 30). Notably, growing evidence suggests that adhesion molecules in the immunoglobulin superfamily including VCAM1 can modulate actin cytoskeleton rearrangement of endothelial cells (31–34). To our knowledge, our report is the first study that demonstrates the role of an adhesion molecule in LSEC capillarization during liver injury. Whether and how aberrantly expressed VCAM1 can cause remodeling of the actin cytoskeleton is a subject of future investigation.

Hippo signaling pathway is an evolutionally conserved pathway, which plays an important role in regulating cell proliferation, organ size, tissue development and regeneration (35). Accumulating evidence suggests the involvement of the Hippo pathway in fibrosis in various organs including lung, heart, pancreas and liver (36–39). YAP1 is a transcriptional coactivator that is negatively regulated in the Hippo signaling pathway. Knockdown of YAP1 expression or pharmacological inhibition of YAP1 prevented HSC activation *in vitro* and pharmacological inhibition of YAP1 ameliorated CCl_4_ or bile duct ligation-induced hepatic fibrogenesis in mice (14). To date, a variety of intracellular signaling pathways including acid ceramidase, fibroblast growth factor 18 and Hedgehog signaling pathways have been shown to act as a modulator of YAP1 activity, thereby altering HSC activation and liver fibrosis (40–42). Interestingly, the adhesion molecule integrin beta-1 (ITGβ_1_) has also been found to play an essential role as an upstream effector of YAP1 regulating HSC activation (43). Given that ITGβ_1_ heterodimerizes with α integrins such as ITGα4 and α7 on the cell surface and VCAM1 is one of the principal ligands for these integrins, ITGβ_1_/YAP1 axis might possibly serve as a key mechanism for VCAM1-induced YAP1 activation and HSCs activation (43). In addition, soluble VCAM1 is released when cleaved by a disintegrin and metalloproteinase 17 (ADAM17) at the extracellular site proximal to the cell membrane (44, 45), then can bind and activate integrin expressed on the target cells (46). Further studies are ongoing in our laboratory to delineate how VCAM1 modulates the Hippo signaling pathway during HSCs activation.

We employed 3 known mouse models of liver injury; each has its advantages and disadvantages. The FFC feeding model phenocopies the histological features and metabolic profiles of human NASH (1); however, it requires 6 months of feeding for histological features to be established. In our preliminary study, we have confirmed that the inducible Cre-mediated endothelial cell-specific gene deletion system we employed can deliver an adequate gene knockout efficacy both in a physiological condition and even after the 6-weeks of CD-HFD feeding. However, gene knockout efficacy after the 24 weeks of FFC feeding was suboptimal, likely secondary to reduced Cre recombinase activity over time after tamoxifen administration. Therefore, we employed CD-HFD diet to induce NASH when using the tamoxifen-inducible endothelial cell-specific gene knockout system. The findings obtained in the mouse NASH model are clinically important since NASH is currently the most common chronic liver disease and a major cause of end-stage liver disease worldwide (47). Furthermore, we demonstrated that inhibition of LSEC VCAM1 can ameliorate not only NASH-related liver fibrosis but also liver fibrosis in a more generalized context by employing CCl4-induced liver fibrosis model. CCl4 is a known model of liver fibrosis caused by hepatocyte necrosis and subsequent liver inflammation arising from the centrilobular area and is broadly used as liver injury model.

Taken together, the findings in the current study suggest that VCAM1 in LSECs is not just a scaffold for leukocyte adhesion, but also a direct modulator of liver fibrosis, further strengthening the potential efficacy of targeting VCAM1 in chronic liver disease patients in clinical settings.

## Data availability statement

The original contributions presented in the study are included in the article/supplementary material. Further inquiries can be directed to the corresponding author.

## Ethics statement

This study was reviewed and approved by the Institutional Animal Care and Use Committee (IACUC) of Mayo Clinic.

## Author contributions

QG: designing research studies, conducting experiments, acquiring data, analyzing data, and manuscript drafting. KF: designing research studies, conducting experiments, acquiring data, analyzing data, and manuscript drafting. NC: designing experiment and discussing data. KD, EK and SI: conducting experiments, acquiring data, analyzing data DJT: manuscript revision. PH: manuscript revision. SHI: concept formulation, designing research studies, analyzing data, and manuscript drafting and revision.

## Funding

Research reported in this publication was supported by the National Institute of Diabetes and Digestive and Kidney Diseases of the National Institutes of Health under Award RO1DK122948 to SHI and P30DK084567 to the Mayo Clinic Center for Cell Signaling in Gastroenterology, and JSPS Overseas Research Fellowships to KF. Support was also provided to PH by the National Institute of Diabetes and Digestive and Kidney Diseases of the National Institutes of Health under Award RO1DK30884 and by Mayo Clinic Center for Biomedical Discovery, and AASLD Pinnacle award and Regenerative Medicine Minnesota research award to EK.

## Acknowledgments

We thank Dr. Gregory J. Gores for his thorough review of the manuscript. We also thank Bing Q. Huang for his help with the electron microscopy studies.

## Conflict of interest

The authors declare that the research was conducted in the absence of any commercial or financial relationships that could be construed as a potential conflict of interest.

## Publisher’s note

All claims expressed in this article are solely those of the authors and do not necessarily represent those of their affiliated organizations, or those of the publisher, the editors and the reviewers. Any product that may be evaluated in this article, or claim that may be made by its manufacturer, is not guaranteed or endorsed by the publisher.

## Glossary

**Table:**

ADAMS17	a disintegrin and metalloproteinase 17
ALT	alanine aminotransferase
aSMA	alpha smooth muscle actin
ANKRD1	ankyrin repeat domain protein 1
BSA	bovine serum albumin
CCl4	carbon tetrachloride
CCR2	C-C chemokine receptor type 2
CD68	cluster of differentiation 68
CD-HFD	choline-deficient high-fat diet
CLD	chronic l iver diseases
CTGF	connective t i ssue growth factor
DAB	diaminobenzidine
DAPI	4′6-diamidino-2-phenylindole
DMEM	Dulbecco’s Modified Eagle Medium
FBS	fetal bovine serum
FFC diet	high fat fructose and cholesterol diet
GAPDH	glyceraldehyde-3-phosphate dehydrogenase;
hLSEC	human liver sinusoidal endothelial cells
HSC	hepatic stellate cells;
hHSC	human hepatic stellate cells
HUVECs	human umbilical vein endothelial cells
IACUC	institutional animal care and use committee
IHL	intrahepatic leukocyte
IL	interleukin
ITG	integrin
JNK	c-Jun N-terminal kinase
LPC	lysophosphatidylcholine
LSECs	liver sinusoidal endothelial cells
MAPK	mitogen-activated protein kinase
mHSC	mouse hepatic stellate cells;
mRNA	messenger RNA
NAFLD	nonalcoholic fatty liver disease
NASH	nonalcoholic steatohepatitis
NF-kB	nuclear factor-kappa B
PA	palmitate;
PAGE	polyacrylamide gel electrophoresis
PBS	phosphate buffered saline;
PCR	polymerase chain reaction
PFA	paraformaldehyde
PDGFR	Plateletderived growth factor receptor
rRNA	ribosomal RNA
SDS	sodium dodecyl sulfate
SEM	standard error of the mean
TIMP1	TIMP metallopeptidase inhibitor 1
TNF-a	tumor necrosis factor-a
VCAM1	vascular cell adhesion molecule 1
VEGF	vascular endothelial growth factor
WCL	whole cell lysate;
WT	wild type
Yap1	Yes-associated protein 1
