# Supplementary material for: Liver sinusoidal endothelial cell expressed vascular cell adhesion molecule 1 promotes liver fibrosis
Source: Front Immunol. 2022 Aug 25;13:983255. doi: 10.3389/fimmu.2022.983255 (PMC9453231; doi:10.3389/fimmu.2022.983255)

Supplementary Material

Liver Sinusoidal Endothelial Cell Expressed Vascular Cell Adhesion Molecule 1 (VCAM1) Promotes Liver Fibrosis

Qianqian Guo[1†], Kunimaro Furuta[2†], Shahidul Islam[1], Nunzia Caporarello[3], Enis Kostallari[1] , Kobe Dielis[1], Daniel J. Tschumperlin[3], Petra Hirsova[1], Samar H. Ibrahim[1, 4*]

[1]Division of Gastroenterology & Hepatology, Mayo Clinic, Rochester, Minnesota, USA

[2] Department of Gastroenterology and Hepatology, Osaka University Graduate School of Medicine, Osaka, Japan

[3]Department of Physiology and Biomedical Engineering, Mayo Clinic, Rochester, Minnesota, USA

[4] Division of Pediatric Gastroenterology, Mayo Clinic, Rochester, Minnesota, USA

Table 1

PCR primers

**Name**	**Sequence**
Mouse *Cd68* Forward Primer	TGTCTGATCTTGCTAGGACCG
Mouse *Cd68* Reverse Primer	GAGAGTAACGGCCTTTTTGTGA
Mouse *Ccr2* Forward Primer	ATCCACGGCATACTATCAACATC
Mouse *Ccr2* Reverse Primer	CAAGGCTCACCATCATCGTAG
Mouse *Col1a1* Forward Primer	GCTCCTCTTAGGGGCCACT
Mouse *Col1a1* Reverse Primer	CCACGTCTCACCATTGGGG
18S Forward Primer	CGCTTCCTTACCTGGTTGAT
18S Reverse Primer	GAGCGACCAAAGGAACCATA

Mouse *Cd34* Forward Primer	AAGGCTGGGTGAAGACCCTTA
Mouse *Cd34* Reverse Primer	TGAATGGCCGTTTCTGGAAGT
Mouse *Timp1* Forward Primer	AGGTGGTCTCGTTGATTTCT
Mouse *Timp1* Reverse Primer	GTAAGGCCTGTAGCTGTGCC
Mouse *Col1a1* Forward Primer	GCTCCTCTTAGGGGCCACT
Mouse *Col1a1* Reverse Primer	CCACGTCTCACCATTGGGG
Mouse *Acta2* (αSMA) Forward Primer	GTCCCAGACATCAGGGAGTAA
Mouse *Acta2* (αSMA) Reverse Primer	TCGGATACTTCAGCGTCAGGA
Mouse *Pdgfrb* Forward Primer	ACTACATCTCCAAAGGCACCT
Mouse *Pdgfrb* Reverse Primer	TGTAGAACTGGTCGTTCATGGGCA
Human *YAP1* Forward Primer	CCCTCGTTTTGCCATGAACC
Human *YAP1* Reverse Primer	TGCCGAAGCAGTTCTTGCTG
Human *CTGF* Forward Primer	GCAGCGGAGAGTCCTTCCAG
Human *CTGF* Reverse Primer	GGGCCAAACGTGTCTTCCAG
Human *ANKRD1* Forward Primer	TGATTATGTATGGCGCGGATCT
Human *ANKRD1* Reverse Primer	GCGAGAGGTCTTGTAGGAGTTC
Human *COL1A1* Forward Primer	TGTGAGGCCACGCATGAG
Human *COL1A1* Reverse Primer	CAGATCACGTCATCGCACAA
Human *TIMP1* Forward Primer	CTTCTGGCATCCTGTTGTTG
Human *TIMP1* Reverse Primer	GGTATAAGGTGGTCTGGTTG

Human *PDGFRA* Forward Primer	TGGCAGTACCCCATGTCTGAA
Human *PDGFRA* Reverse Primer	CCAAGACCGTCACAAAAAGGC
Human *PDGFRB* Forward Primer	AGCACCTTCGTTCTGACCTG
Human *PDGFRB* Reverse Primer	TATTCTCCCGTGTCTAGCCCA

Supplementary Figure 1. ***Liver sinusoidal endothelial cells Lyve1 expression is reduced in liver injury.*** Quantification of immunostaining of Lyve1 on liver sections from (A) CD-HFD induced NASH mouse model and (B) CCl4 induced liver fibrosis mouse model. Scale bar: 50µm, n=5-6. (C)Representative images of co-immuno staining of Lyve1 and αSMA in liver sections from CD-HFD or chow fed mice. Scale bar: 10 µm, left & middle panels. 5 µm, right panel, n=3.

Supplementary Figure 1

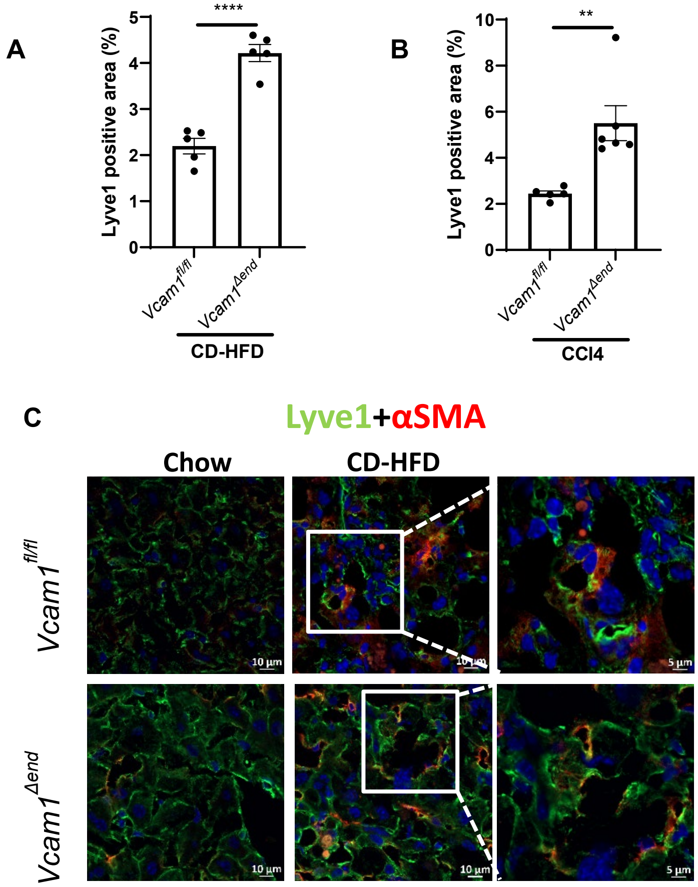

Supplementary Figure 2: (A) hHSCs were treated with vehicle or 5 ng/ml of human recombinant TGFβ for 16 hours. Protein expressions of fibronectin and α-SMA were examined by Western blot. Heat shock cognate 70 (HSC70) was used as a loading control.

A

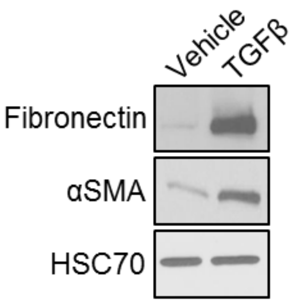

Supplement: Supplementary file 1 [file DataSheet_1.pdf]
